# Fabrication and Characterization of a Porous TiO_2_‑Modified PEEK Scaffold with Enhanced Flexural Compliance for Bone Tissue Engineering

**DOI:** 10.1021/acsbiomaterials.5c01032

**Published:** 2025-09-23

**Authors:** Martina Galea Mifsud, Andrew Sachan, Roger J. Narayan, Lucy Di-Silvio, Trevor Coward

**Affiliations:** a Faculty of Dentistry, Oral & Craniofacial Sciences, 388741 King’s College London, London SE1 9RT, U.K.; b Joint Department of Biomedical Engineering, 6798 University of North Carolina and Carolina State University, Raleigh, North Carolina 27695-7115, United States

**Keywords:** bone tissue engineering, 3D printing, polyetheretherketone scaffold, titanium dioxide coating, biomaterial characterization, mechanical properties

## Abstract

Bone pathologies are becoming increasingly prevalent with an aging population, often necessitating bone grafting procedures. The current gold standard for grafting uses autologous tissue; however, this approach carries limitations such as donor site morbidity. Consequently, there is a growing interest in alternative biomaterials. Polyetheretherketone (PEEK), a thermoplastic with bone-like mechanical properties, has shown promise, although its limited bioactivity remains a critical constraint. Various functionalization strategies have been employed to enhance the biological performance of otherwise inert materials. This study aims to develop a functionalized porous PEEK scaffold to improve bioactivity of the material, thereby promoting human osteoblast (HOB) adhesion, proliferation, and differentiation. PEEK scaffolds were fabricated using fused deposition modeling (FDM) (Apium P155), with a rectilinear pattern alternating at +45° and −45° angles between layers. This configuration generated an interconnected pore network with sizes ranging from ∼100 to 400 μm. The scaffolds were further coated with titanium oxide as an additional intervention to enhance bioactivity. Mechanical properties of both porous and solid constructs were evaluated according to ISO 178, a flexural testing standard for plastics. Results indicated that both porous scaffolds exhibited a 10-fold decrease in flexural modulus and were 10 times more flexible compared to the solid counterpart (*p* < 0.001). The mechanical properties of both porous scaffolds were consistent with values reported for trabecular bone, while the solid construct demonstrated a flexural modulus comparable to cortical bone. These findings suggest that the porous PEEK scaffold, both neat and titanium oxide-coated, possesses mechanical properties similar to bone *in vivo*, indicating its potential as a mechanically suitable biomaterial for bone grafting applications.

## Graphical abstract

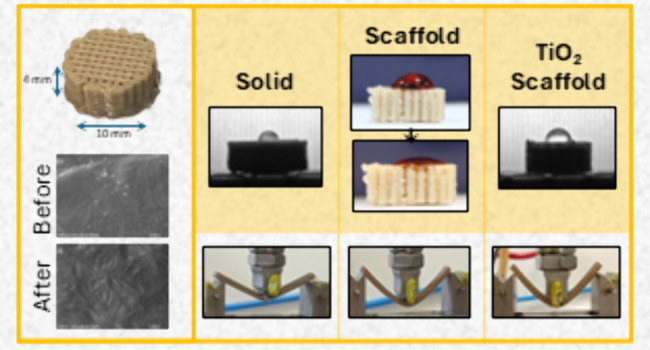

## 1 Introduction

With an increasingly aging population, medical advancements are essential to meet evolving healthcare needs. This is particularly true in osteology, in which bone defects and traumata may arise congenitally, through tumor development and subsequent ablation, or from pathological conditions such as osteoporosis. Among these challenges, critical-sized bone defects, which are described as defects that “will not heal spontaneously within a patient’s lifetime”,^1^ typically with a “length greater than 1–2 cm”^2^ present significant clinical hurdles and often require surgical intervention, such as bone grafting.^3^

Autologous bone grafting has long been the gold standard for treating such defects.^3^ However, limitations including availability, unpredictable resorption during healing, and donor site comorbidity^4^ have driven researchers to explore alternative solutions. One promising approach involves the development of alloplastic grafts for tissue engineering, which offer fewer inherent limitations and have demonstrated significant potential.^5^

Among the various biomaterials being investigated, polyetheretherketone (PEEK) is a notable candidate. PEEK is a high-temperature thermoplastic polymer that exhibits favorable physical and chemical properties that are suitable for bone grafting applications. Its mechanical properties have been shown to closely match those of bone.^6^ Chemically, PEEK is an inert compound that does not elicit cytotoxic or mutagenic reactions *in vitro* ^7^ and demonstrates very low solubility in water,^8^ making it well-suited for application in the human body’s biochemical environment. Additionally, it has a relatively high glass transition temperature *T*_g_ of 145 °C, below which it remains glassy, hard, and dimensionally stable.^9^ These properties render it suitable for long-term use in the human body, which is a dilute environment at 37 °C.^10^

However, a significant drawback of this material is its inherent bioinertness, which hinders satisfactory wetting and cell adhesion—critical steps in bone healing. Addressing this limitation typically involves surface chemical modifications or alterations to the material body, both chemically and physically.^11^ This current study focuses on geometrical modifications to the material body, as well as a tertiary coating of titanium oxide, with the resulting samples evaluated according to ISO 178 standards for flexural testing of rigid plastics. This is because flexural forces are present in the maxillofacial area as exemplified by mandibular flexure,^12^ which is one of the intended uses of the PEEK scaffold.

Apart from composite fabrication, geometrical modifications to PEEK can be exploited through an interconnected network of pores. The presence of pores within a construct intended for use in bone grafting is one of the main requirements of the ideal bone graft substitute. In fact, an ideal construct should have adequate porosity, adequate interconnectivity, the strength of the material and the resorption ability of the material.^13^ The ideal structure should [i] have an interconnected porosity (to allow cell diffusion) of adequately sized pores (to allow cell attachment and vascularisation).^14^ Although the optimal pore size for orthopedic scaffolds remains unclear, a minimum of ∼100 μm ^15^ and a maximum exceeding 300 μm,^13^ potentially up to 600 μm ^16^ are recommended to ensure sufficient vascularisation. Larger pores are preferred for cell growth and proliferation as pore occlusion will occur later compared to smaller pores, thereby providing a more open, interconnected space for oxygen and nutrient supply because of the enhanced vascularisation.^16,17^ Another important aspect of such a biomaterial is that it should [ii] allow vascular ingrowth enhanced by its interconnectivity, facilitating osteogenic cell adhesion, migration and proliferation. While properties conducive to the construct’s bioactivity are important, it is just as crucial that the material is [iii] sufficiently strong in terms of its compressive and elastic abilities, giving rise to load sharing. The resultant structure will ideally mimic a solid body of PEEK in terms of its physical, chemical and biological properties. Another requirement is for it to be [iv] resorbable, with the rate of resorption ideally the same as that of bone ingrowth, and the last requirement is that it should have sufficient dimensional stability to withstand chairside adaptation of the bone graft on site during the intervention.^13^ PEEK is a nonbiodegradable material, and while this could be seen as a shortcoming, permanent scaffolds have their advantages as they remain unchanged over time, providing permanent stability throughout the patient’s lifetime.^18^ The chosen method of fabrication of PEEK in this study was Fused Deposition Modeling (FDM). PEEK is notoriously challenging to additively manufacture due to a number of factors.^19^ These comprise its high printing temperature^20^ the resulting large thermal gradient causing warpage and deformation during cooling,^21^ lower accuracy in increasingly complex geometries,^22^ and fast crystallization rate, which could lead to shrinking because of the rearrangement of the PEEK molecules within the construct resulting in a more compact structure.^23^

Apart from modifying the geometry to improve a graft’s bioactivity, surface modifications such as tertiary coatings are commonly used to improve the bioactivity of a PEEK construct.^11^ Several studies have investigated coatings such as graphite oxide, degradable polymers and metals and their oxides,^24^ as well as calcium phosphate and hydroxyapatite depositions^25^ among a vast variety of interventions. Notably, titanium, and by extension, titanium oxide, has also been trialled as a coating for PEEK; significantly increasing osteoblast adhesion.^26^ This oxide layer forms when titanium (Ti) is exposed to a physiologic environment, reacting with oxygen to create an oxide layer with the chemical formula TiO_2_. This gives Ti its biocompatibility property, as apart from being corrosion resistant, it has been proven to absorb phosphate and calcium ions from the bone, suggesting that there is an active exchange of these ions present at the implant-bone interface^27^ which contribute to and expedite osseointegration.^28^ To note, however, that implants composed of commercially pure titanium or even titanium alloys such as Ti-6Al-4 V have a different Young’s modulus to bone^29^ , which difference leads to stress shielding. This is when the bone around the implant atrophies, leading to loosening of the implant and bone resorption.^30^ Therefore, a number of studies have tried to combine the bodily advantages of PEEK, with the biological advantages of a Ti/Titanium oxide coating to create the optimal bone scaffold.^6,31–34^ Torstrick et al.^6^ compared a porous PEEK scaffold to a solid PEEK substrate coated with titanium and found that the porous neat PEEK contributed to increased osseointegration *in vivo* when compared to both smooth PEEK and titanium coated PEEK. The advantages of porous PEEK are clear, however, while titanium oxide coatings have demonstrated benefits on solid PEEK substrates,^6,31,32,34^ their application to porous scaffolds has been reported only sparingly. Available studies indicate that titanium coating on porous PEEK enhances cell attachment and upregulates osteogenic genes expression.^33^

Another argument in favor of titanium modification is the mitigation of the so-called PEEK-halo effect; a complication associated with PEEK implants. Owing to its inferior biocompatibility, PEEK tends to produce a “PEEK-Halo” effect on CT scans, reflecting poorer integration with the host bone^35^ (Figure 1a). By contrast, when titanium is introduced either as a composite component or as a coating on PEEK, this “halo” effect is not observed, with steady bone in-growth observed instead (Figure 1b).

**Figure 1:**
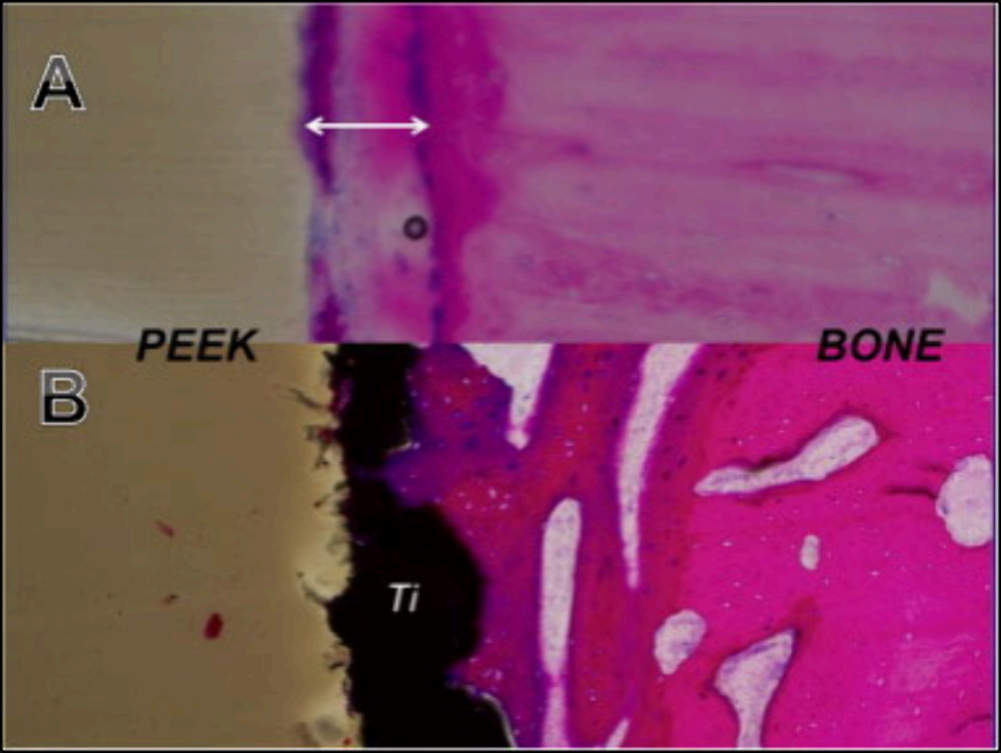
“Histology of PEEK/bone interface at 4 weeks post-implantation into a sheep tibia model. (A) Presence of fibrous tissue (white arrow) between the PEEK implant and adjacent bone—the rim of fibrous tissue results in the halo effect seen on CT imaging. (B) Titanium (Ti)-PEEK/bone interface demonstrating on-growth and ingrowth of bone at the Ti-PEEK/bone interface, with no radiolucent rim evident on CT imaging.” Reproduced with permission from ref 35, Phan ; , 2016; The Journal of Clinical Neuroscience . Copyright License 6073041362909 Elsevier.

Taken together, current evidence highlights porous PEEK as a highly promising scaffold material for bone tissue engineering, particularly when combined with surface modifications to overcome its inherent bioinertness. Within this context, TiO_2_-modified porous PEEK presents a compelling candidate for experimental investigation, with its fabrication and subsequent characterization providing critical insights into its structural and functional potential.

## 2 Materials and Methods

### 2.1 Fabricating the Scaffold

The study focused on the fabrication and characterization of a porous scaffold made of PEEK using an Apium P155 filament 3D FDM printer (Apium Additive Technologies GmbH, Karlsruhe, Germany). The criterion for the final construct was to include controllable pores and channels in terms of size, creating an interconnected meshwork of pores that would allow for cellular infiltration. This porous scaffold was then coated with titanium oxide (TiO_2_). The three constructs used throughout this study were therefore solid PEEK as a control (C1), untreated neat PEEK scaffold (S1) and titanium oxide coated scaffold (S2). The methods for fabricating each construct are discussed below.

For the solid construct, cylinders of 10 mm diameter and 4 mm height were successfully designed in Autodesk Inventor (R) (Autodesk (R), San Francisco, USA), sliced in Simplify3D (R) (Simplify 3D, Cincinnati, USA), and printed on the Apium P155 FDM printer using Apium PEEK 450 1.75 mm (both Apium Additive Technologies GmbH, Karlsruhe, Germany) (Figure 2). The five samples in the figure are surrounded by a ‘skirt’ and a ‘raft’ – parts that were added to help anchor the print to the bed to improve the chances of a successful 3D printed build. Without the skirt and/or raft, a print may detach from the bed, leading to a failed print, notwithstanding the correct bed temperature.

**Figure 2:**
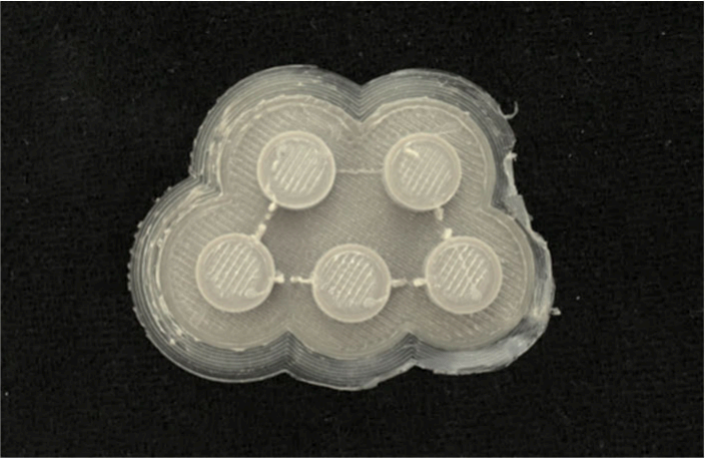
FDM fabricated PEEK solid samples (C1). The diameters are 10 mm (W) by 4 mm (H).

The porous construct was also designed as a solid cylinder in CAD. The slicing software Simplify3D was used to create the porous geometry with key parameters 50% infill density with a rectilinear printing pattern at alternating layers (+45 °C, −45°). This approach successfully produced a porous geometry due to its inherently porous repeated unit (S1). Using these parameters, the scaffold was confirmed to have pore sizes of 100–400 μm width, fulfilling the criteria stipulated for a tissue engineering scaffold. Further details on the fabrication of these scaffolds can be found in our previous publication.^63^

### 2.2 Modifying the Scaffold

For the unfilled construct, the printed scaffold was left intact. For modification of the scaffold with the titanium oxide (TiO_2_) coating (S2), atomic layer deposition (ALD) was carried out in a Veeco/CNT Savannah S200 thermal ALD system (Plainview, NY, USA) using tetrakis­(dimethylamino)­titanium­(IV) [[(CH_3_)_2_N]_4_Ti, TDMAT, >99.9%) and distilled water (H_2_O) as the precursors. Research-grade nitrogen (N_2_) was used as both the carrier and purge gas. The substrate temperature was maintained at 150 °C, while the precursor manifold and exhaust trap were maintained at 130 °C. The TDMAT and H_2_O precursors were held at 75 °C and room temperature, respectively. To ensure more complete conformal deposition on the high aspect ratio features of the substrate, exposure (expo) mode was employed. In expo mode, the reactor is isolated from the roughing pump while the precursor is delivered. This approach allows the sample to be exposed to the precursor for an extended time, and enables the precursor to diffuse into high aspect ratio structures. The TDMAT and H_2_O precursors were pulsed for 0.3 and 0.02 s, respectively. An exposure time of 5 s and a purge time of 10 s were used for both of the subcycles. The approximate growth rate per cycle was 0.47 Å/cycle, resulting in a 10 nm layer of titanium oxide within the PEEK scaffolds.

### 2.3 Confirming the Titanium Oxide Coating

Scanning Electron Microscopy (SEM), energy dispersive spectroscopy, and X-photoelectron spectroscopy data were obtained from the uncoated and coated scaffolds. SEM imaging was undertaken using a S-4700 field emission microscope (Hitachi, Tokyo, Japan). The samples were positioned on the surface of a double-stick carbon tape on an aluminum SEM sample holder. The as-prepared PEEK samples (S1) were coated using a 5 nm layer of gold–palladium (AuPd) alloy that served as a conductive coating. The S2 samples were not coated with AuPd alloy prior to imaging. A 2 kV accelerating voltage, a 10 μA beam current, and a working distance of close to 12 mm were utilized for SEM analysis. Energy dispersive spectroscopy (EDS) analysis was performed on an INCA PentaFet-X3 instrument (Oxford Instruments, Abingdon, United Kingdom) that was attached to the microscope; an acceleration voltage of 20 kV was used for analysis.

X-ray photoelectron spectroscopy (XPS) data were obtained using a Kratos Supra+ instrument (San Diego, CA, USA) using a monochromatic Kα X-ray source that was operated at 150 W. A charge neutralizer was utilized to prevent charging when necessary; all the spectra were corrected using the C 1s peak at 284.6 eV. Survey scans were obtained at pass energies of 80 and 20 eV, respectively; the analyzed spot size was 300 μm × 700 μm. Kratos ESCApe software (San Diego, CA, USA) was utilized for data analysis, and Shirley baselines were utilized; the relative sensitivity factors used for quantification were those from the ESCApe software.

### 2.4 Physical Tests: Percentage Porosity

The percentage porosity and water absorption of the scaffolds were assessed. In the literature, the main approach to confirming the porosity of a construct is to take the mass, divide by the volume and multiply by the density of the material. The equation (eq 1) used these values to work out how much of the final scaffold was void, thereby giving the percentage of the porosity of the printed construct. Three samples of both the S1 and S2 samples were assessed and the results averaged. Equation 1 illustrates working out the porosity of a scaffold:^36^

1 $$\left[1-\frac{m_{\mathrm{scaffold}}}{v_{\mathrm{scaffold}}}/\rho \mathrm{PEEK}\right]\times 100\%$$

### 2.5 Physical Tests: Water Absorption

Water absorption assesses the amount of water retained by the scaffold over a period of time. Three separate S1 and S2 samples were weighed when dry, then immersed in distilled water for 2 h, and then weighed again.^37^ The scaffolds were then left for a further 22 h (making up 24 h in total) for more long-term immersion^38^ and weighed again to assess any differences. The results were then substituted in the water absorption equation (eq 2), where W_s_ = wet scaffold, and W_d_ = dry scaffold:^39^

2 $$\mathrm{water uptake}\left(\%\right)=\left[\frac{W_{s}-W_{d}}{W_{d}}\right]\times 100$$

### 2.6 Physical Tests: Wettability via Static Water Contact Angle

The wettability was also assessed by pipetting 10 μL of ultrapure water^40^ on the surface of the constructs. Three samples of each of the different surfaces compared (C1, S1 and S2) were prepared, and the static contact angle was taken using a dedicated set up and phone camera (Honor 90 Lite). After adding the drop of ultrapure water, sequential images were captured and analyzed using ImageJ software^41^ with the ‘Contact Angle’ plugin following the methodology described by Buahom (2018).^42^

### 2.7 Physical Tests: Differential Scanning Calorimetry

Differential Scanning Calorimetry (PerkinElmer Jade DSC, Waltham, MA, USA) was used to determine any change in the glass transition temperature before and after processing at high temperatures. Therefore, DSC was conducted on one sample of off-the-shelf PEEK filament and one sample of the processed/printed PEEK filament each weighing approximately 10 mg. Both samples were respectively encapsulated in the appropriate aluminum pans, and after two cycles of heating to 110 °C and cooling to −10 °C to evaporate any water and erase the thermal history. The samples were then taken up to 400 °C at a rate of 10 °C per minute for the phase examination curve, after which the samples were cooled to 30 °C at a rate of 100 °C per minute for rapid cooling to complete the test. This determined any effect of the printing procedure on the glass transition temperature of PEEK before and after processing.

To calculate crystallinity, it was first noted that the software output was plotted automatically as ‘Heat Flow Endo Up’, meaning that the higher along the *y* axis, the more endothermic a reaction is, with the values for heat flow also increasing. However, melting is an endothermic process and so ΔHm, which is the enthalpy of melting, should be a positive, upward peak rather than a downward peak. Since it was automatically plotted by the software as a downward peak, absolute values were used to contextualise the melting phase values. To work out the percentage crystallinity, baseline correction was carried out using formula = $B$N + (($B$M - $B$N)/($A$M - $A$N)) * (A_i - $A$N) where N was the initial anchor point for the start of the endothermic reaction and M was the last anchor point, thereby creating a baseline in between, with A= temperature and B = measured heat flow. The trapezoidal rule was employed to calculate the area underneath the peak, and thus the ΔHm was identified for both samples. To note that all units were corrected and the values mass normalized. The resulting ΔHm was then divided by 130 J/g using the correct equation (eq 3, calculating the percent crystallinity) which is the identified reference enthalpy value for 100% PEEK.^43^

3 $$X_{C}=\frac{\Delta H_{m}}{\Delta H_{m}^{0}}\times 100\%$$

### 2.8 Flexural Testing According to ISO 178

The preferred test specimen for ISO 178 is a cuboid of dimensions 80 × 10 × 4 mm, and five such samples times three interventions (C1, S1 and S2) were additively manufactured using the previously optimized parameters,^63^ and tested (Bose ElectroForce 3330, TA Instruments, New Castle, DE, USA) using the ideal test speed of 5 mm/min. Using these parameters, five samples each of C1, S1, and S2 were tested either until failure or up to the maximum confines of the flexural machine used (3kN, or 20 mm displacement). The results were plotted, and the flexural moduli assessed according to the gradient of the linear portion of the plotted scatter plots—this was done by eluting the equation of the graph then substituting flexural strain εf1 = 0.0005 and εf2 = 0.0025 as per the ISO standard. Statistical analysis was carried out using a one-way ANOVA, employing Tukey HSD and Bonferroni posthoc tests to identify the direction of the effect,

## 3 Results

### 3.1 Scanning Electron Microscopy Imaging

Figure 3 (a–d) shows SEM images of S1 that were obtained at several magnifications. Figure 4 (a–d) shows SEM images of S2 that were obtained at several magnifications. In order to distinguish elemental composition between the coated and uncoated scaffolds, energy-dispersive X-ray analysis was performed. Energy-dispersive X-ray spectra indicated that only carbon and oxygen were present in the spectra of the neat PEEK scaffold; in contrast, carbon, oxygen, titanium, and a trace amount of potassium (0.4%) were identified in the spectra of S2. Figures S2A, S2C, and S2E within the Supporting Information shows the SEM images obtained at Site #1, Site #2, and Site #3, respectively, on the titanium coated PEEK scaffold. Figures S2B, S2D, and S2F also within the Supporting Information show the energy dispersive spectroscopy data corresponding to Site #1, Site #2, and Site #3, respectively. The energy dispersive spectroscopy data shown in these images and Table S1 (corresponding to Site #1), Table S2 (corresponding to Site #2), and Table S3 (corresponding to Site #3) indicate the presence of titanium on the titanium coated PEEK scaffold. No trace levels of other elements were noted.

**Figure 3:**
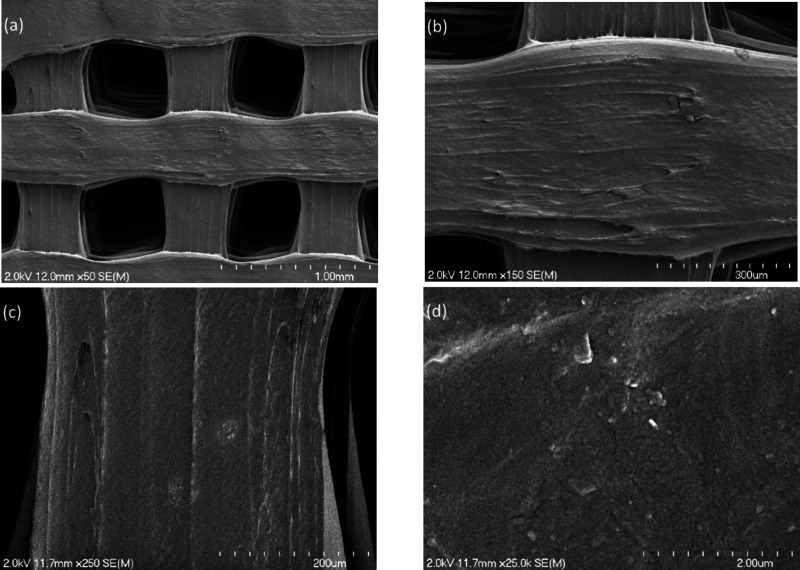
(a–d) SEM images of S1 that were obtained at several magnifications, prior to coating.

**Figure 4:**
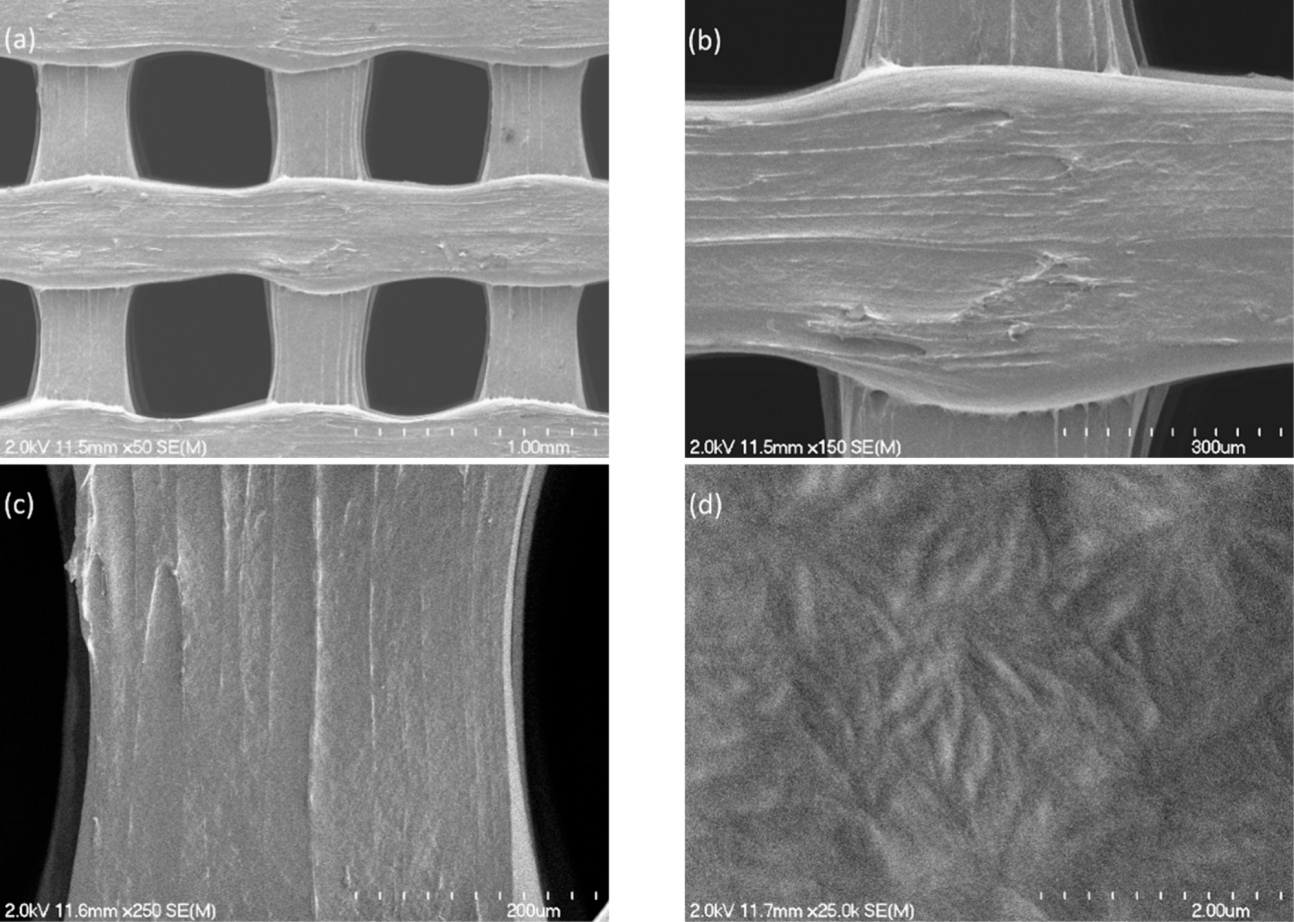
(a–d) SEM images of S2 that were obtained at several magnifications.

### 3.2 X-ray Photoelectron Spectroscopy Chemical Analysis

X-ray photoelectron spectroscopy is widely used to evaluate the chemical composition of different constructs;^44,45^ and in this case S1 and S2. The atomic concentration of S1 was noted to be 85.3±0.1% for carbon and 14.7±0.1% for oxygen; no other elements were noted (Figure 5a). The atomic concentration of S2 was noted to be 56.9 ± 0.3% for oxygen, 25.5 ± 0.1% for titanium, 17.2 ± 0.7% for carbon, and 0.4 ± 0.0% for potassium; no other elements were noted (Figure 5b).

**Figure 5:**
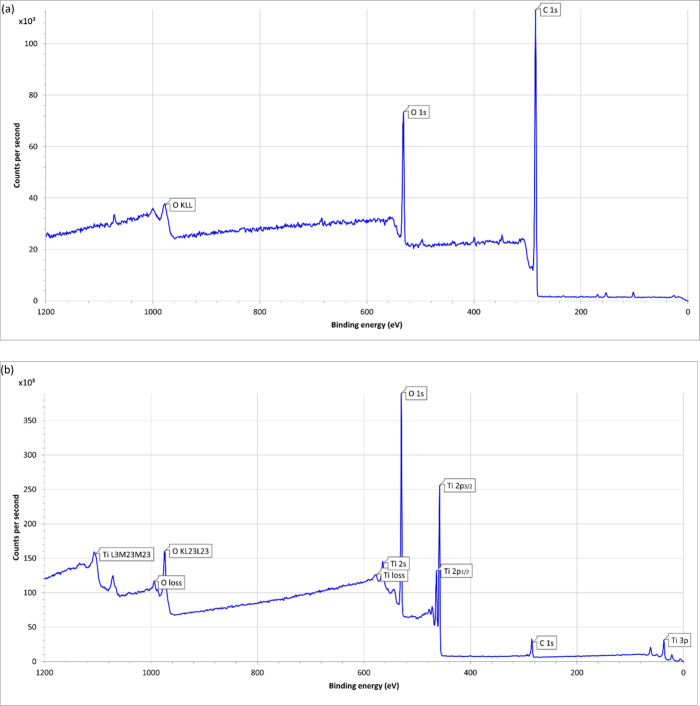
(a) X-ray photoelectron survey spectrum of a neat PEEK scaffold. (b) X-ray photoelectron survey spectrum of S2.

### 3.3 Physical Test: Percentage Porosity

Porosity of the construct was confirmed by dividing the mass by the volume, and the result multiplied by the density of the material. Three separate S1 and S2 samples were assessed and their measurements averaged. For S1, the final value confirmed that the printed construct (51.03% porosity) was an almost ideal representation of the designed construct (50% porosity). The final S2 was slightly less porous, at 48.43%. This result can be attributed to the titanium oxide coating within the scaffold, occluding the pores due to its 10 nm thickness. Other differences were therefore most likely attributable to printing errors.

### 3.4 Physical Test: Water Absorption

The weights of three S1 and S2 samples were taken before and after 24 h of immersion in water; averaged and substituted in the water absorption equation. The result showed that S1 can uptake up to 57.4% of its volume, while S2 can uptake up to 53.09% of its volume. Like the porosity results, this difference between S1 and S2 can be attributed to the reduced pore sizes within the scaffold because of the titanium oxide coating.

### 3.5 Physical Test: Wettability via Static Water Contact Angle (WCA)

A drop of water behaves differently on a solid surface depending on how wettable the surface is.^46^ The 10 μL drop of water^40^ behaved differently on both scaffolds S1 and S2 compared to C1 (Figure 6 a–c). The mean water contact angle for C1 was 86.30° (SD ± 12.74), for S1 was 77.40° (SD ± 46.87) and for S2 was 96.93° (SD ± 9.53). After half an hour of exposure, the C1 water contact angle remained unchanged, however S1’s water contact angle became 83.45° (SD ± 14.99) and S2’s contact angle became 75.57° (SD ± 2.21). Given the high standard deviation observed especially for the untreated scaffold samples S1, additional samples would need to be tested both to reduce variability and to better understand why the contact angle changed unfavorably for the untreated scaffold S1. Notwithstanding this, it is worthwhile noting that both scaffolds attained a hydrophilic contact angle after just half an hour, making this construct the ideal substrate for cell adhesion further along the project pathway. In fact, in a separate technical repeat, the untreated scaffold S2 showed a drastic decrease in contact angle (Figure 6 d, e) also after 30 min, indicating that the scaffold does have hydrophilic tendencies. Although the contact angle on the solid PEEK C1 appeared more hydrophilic, accurate measurements were not possible because the 10 μL drop spread along the crests of the surface topology. This spreading also explains the seemingly reduced droplet volume in the image.

**Figure 6:**
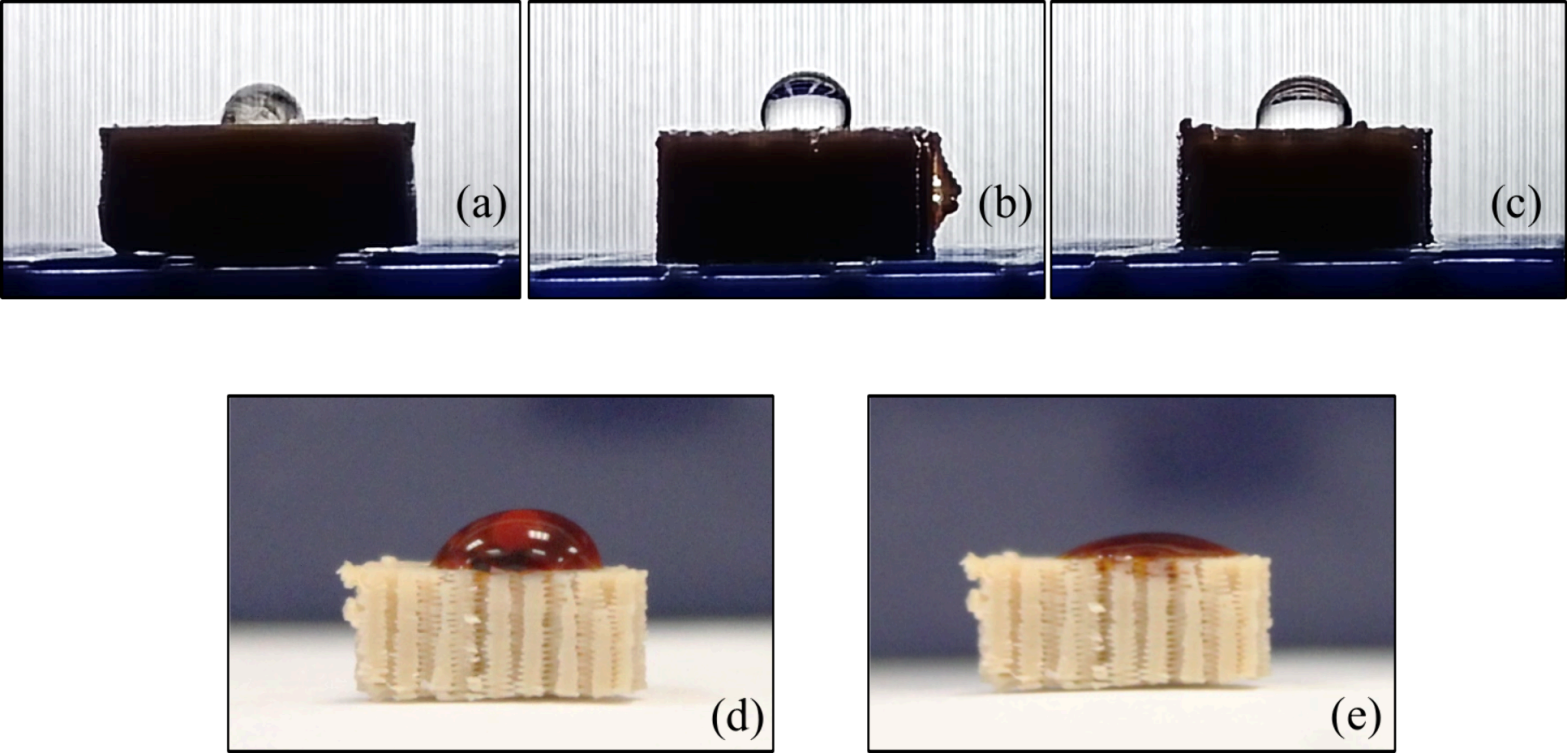
WCA on the surface of (a) C1, solid PEEK disc; (b) S1, a 50% porous PEEK scaffold; (c) S2, a titanium oxide coated 50% porous PEEK scaffold. (d) A colored drop of ultrapure water on untreated scaffold PEEK, photo taken immediately and after half an hour (e).

### 3.6 Physical Test: Differential Scanning Calorimetry

The numerical data obtained from the DSC was plotted, and the *T*_g_ was calculated from the relevant section on the graphs. For the off-the-shelf filament PEEK, the glass transition temperature was 160.41 °C, and for the processed/printed PEEK, the glass transition temperature was 155.03 °C, analyzed using the inbuilt software heatflow trace. The percentage crystallinity was 16.49% crystallinity for the unprinted sample with a ΔHm of 0.2047J, and 10.92% crystallinity for the printed at 435 °C sample, with a ΔHm of 0.1555J.

### 3.7 Mechanical Test: ISO 178 Three-Point Bend Test

Five samples per intervention were used to assess the flexural properties of both S1 and S2 when compared to C1 (Figure 7 a–c). The resultant values processed into flexural stress and strain were plotted together for C1 (Figure 8a), S1 (Figure 8b) and S2 (Figure 8c), showing primarily the different range of stress on the material and second that C1 was tested until failure, whereas both versions of the PEEK scaffold were only tested to the maximum confines of the machine (3 kN). The average flexural modulus for C1 was 2.37 GPa, and 0.21 GPa (213.81 MPa) for S1; this 10-fold decrease observed construct indicates that the porous construct is significantly more flexible. The S2 samples behaved similarly to S1, with a flexural modulus of 201.3 MPa. It was interesting to note that S1 did not undergo any form of permanent deformation as it reverted to its original shape upon test completion, unlike S2, which was permanently deformed after the testing.

**Figure 7:**
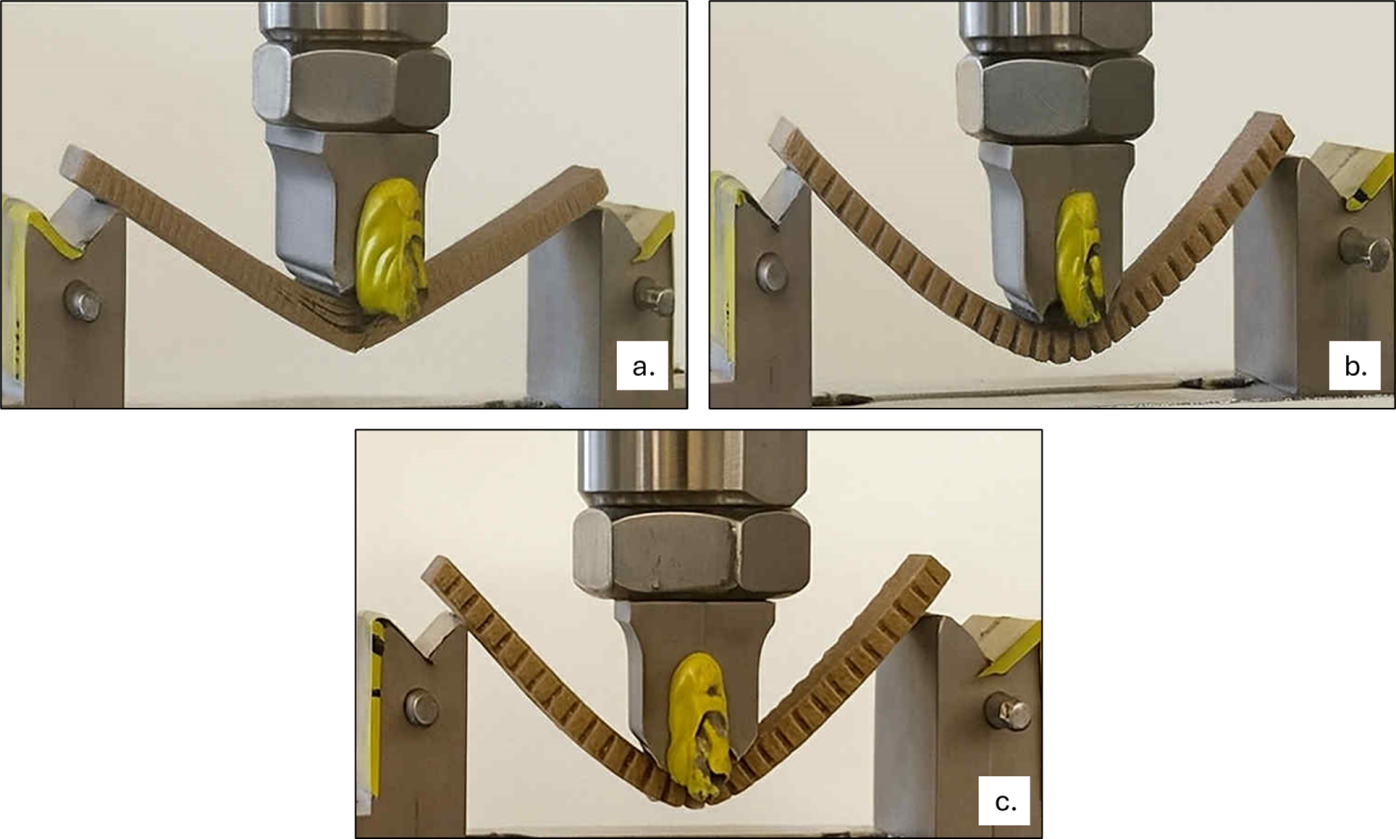
ISO 178 3-point bend test. (a) C1 flexural testing. The construct snapped before the test reached its maximum displacement. (b) S1 testing, with the construct flexing then returning to its original shape upon test completion. (c) S2 flexural testing. The scaffold did not fracture; however, it was permanently deformed.

**Figure 8:**
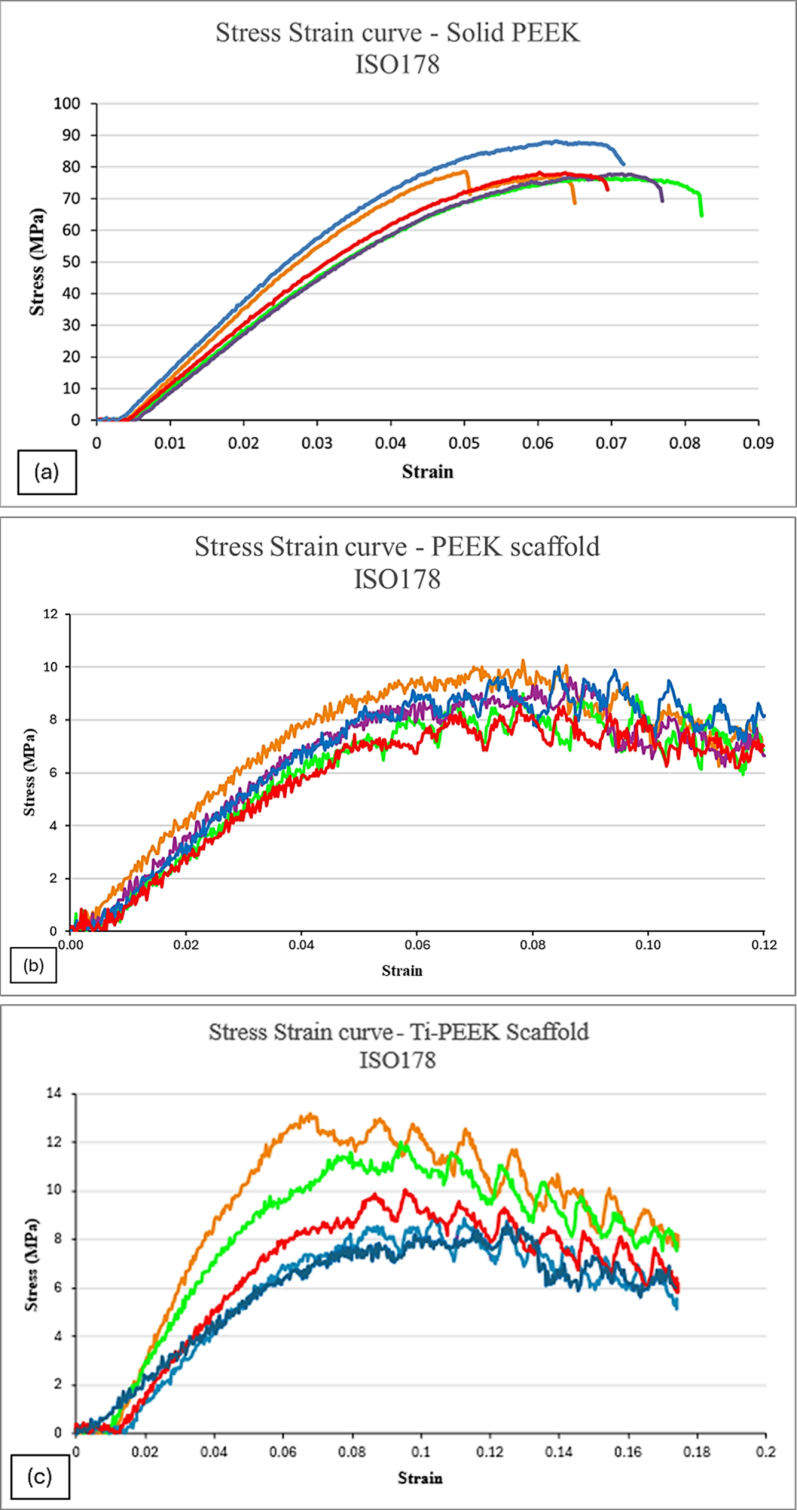
(a) Flexural stress strain curves for five C1 samples. (b) Flexural stress strain curves for five S1 samples. (c) Flexural stress strain curves for five S2 samples.

C1 had a flexural modulus of 2369.8 MPa, S1 had a flexural modulus of 213.81 MPa, and S2 had a flexural modulus of 201.3 MPa. A statistically significant difference was observed between the three groups, with p = <0.001 with no statistically significant difference between both types of scaffold (S1 and S2) groups (p = 0.986 and p = 1.000 for both posthoc tests respectively) but there was a highly statistically significant difference between C1 when compared to both types of scaffold (p = <0.001), confirmed by both posthoc tests.

This finding indicates that C1 was most resistant to flexural forces ultimately fracturing under excess flexural forces; but the flexibility increased significantly with S1 and S2.

## 4 Discussion

The fabrication of a porous scaffold for use in bone tissue engineering was essential to support cell infiltration, nutrient diffusion, and tissue integration, marking key progression from previous work with solid constructs. The current work builds on this prior experience to create a more biologically functional scaffold architecture.

Once a porous scaffold was successfully optimized and fabricated via FDM,^63^ titanium dioxide was the chosen modification to improve the biocompatibility of PEEK, due to its proven osteogenic potential, as well as improved *in vivo* behavior over untreated PEEK (Figure 1).^6,28,31–35^ This was applied to the PEEK substrate through ALD which forms uniform coatings on all exposed surfaces of the scaffold—in fact, this attribute makes ALD an attractive approach to chemically modifying the surfaces of biomedical materials. Cameron et al.^47^ previously demonstrated that saturation of the individual surface reactions during the atomic layer deposition process involving metal oxide materials (e.g., titanium oxide) provides nearly ideal film growth in a layer-by-layer manner.^47–49^ This was evident in the fairly uniform SEM contrast noted on the functionalized S2 in Figure 3; no pinholes or other nonuniformities in the titanium oxide coating were observed. Atomic layer deposition is capable of forming conformal and uniform coatings on all exposed surfaces of the scaffold. When analyzing the results from the X-ray photoelectron spectroscopy (Figure 5b), a small percentage (0.4 ± 0.0%) of potassium was found to be present. No other elements (e.g., elements of known toxicity) were noted in the X-ray photoelectron spectrum of the coated scaffold. The source of the potassium contamination is unclear; sources of contamination will be assessed and minimized prior to future studies.

Once the scaffold was fabricated and coated with TiO_2_, confirming its mechanical and physical suitability for use in bone grafting was necessary. A variety of tests were chosen. Initially, the water contact angle assessment was selected as it directly reflects the wettability of a material, which ensures the success of cell adhesion which is a first step for successful osseointegration down the line. Interestingly, a noticeable change in the water contact angle was observed between the solid PEEK disc and the PEEK scaffold, which was accentuated after just 30 min (Figure 6). Even without surface treatment, the scaffold’s geometry alone enhanced its hydrophilicity when compared to its solid counterpart. Increased hydrophilicity, or wettability facilitates better cell adhesion, thereby promoting osseointegration.^50^ The reduction in water contact angle observed could have also been attributable to microlevel surface roughness, however, given that the scaffold was fabricated via filament extrusion, the formation of such roughness was minimal. This is due to the high shear rates present during printing, and the tendency for molecular relaxation during the process to smooth out any present inhomogeneity or defect.^53^ Therefore, the reduction in water contact angle between the solid and scaffold PEEK is likely attributable to the macroporous structure of the scaffold. The *xy* channels through which the drop of water flowed cohesively, have an effect on the shear stress present.^51^ Fluid shear stress has already been confirmed to promote differentiation of different cell types,^52^ which would be beneficial for osteogenesis and osseointegration of the scaffold. Overall, scaffolds such as the ones fabricated in this study can exhibit greater inherent hydrophilicity, due to their increased surface area, improved wettability or their surface roughness.^53^ However, a larger sample size is necessary to confirm any significant reduction in water contact angle and thus hydrophilicity.

Differential scanning calorimetry showed that when processed, the physical properties of the material were slightly altered. The lowered crystallization of the semicrystalline thermoplastic after cooling from the printing temperature of 435 °C might have influenced the *T*_g_; further work would need to be carried out to better understand this phenomenon. The percentage crystallinity of unprocessed PEEK filament was 16.49% for the unprinted sample, and 10.92% crystallinity for the printed at 435 °C sample. This is relatively low, in light of the fact that PEEK is considered to be a semicrystalline thermoplastic. PEEK is recognized to be amorphous when molten, but is able to crystallize; or form crystalline structures during its solidification. The type and shape of these structures can depend on conditions such as cooling rate, melt pressure and flow history.^54^ The molecular weight of the PEEK used can also have a significant impact on its crystallinity—in fact, a higher molecular weight has been linked to lower degrees of crystallinity. A relatively high molecular weight could possibly contribute to the lower glass transition temperature and the lower crystallinity. This inverse relationship between molecular weight and crystallinity has already been noted in the literature, which correlates the longer polymer chains with an increased difficulty in molecular reorganization during cooling, leading to a lower degree of crystallinity.^54^ This extent or degree of crystallinity can be adjusted with control of the ambient temperature during the printing process,^55,56^ as exemplified in more advanced 3D printer models which have a heated printing chamber.

Both solid and scaffold interventions were then tested mechanically according to the flexural testing standard ISO 178 with *n* = 5 sample size. This guideline was used to assess the flexural properties of S1 and S2 when compared to C1. To note that this standard was chosen in the absence of a better standard for polymers that have been FDM/additively manufactured.^57^ Ideally, this standard is not used for plastics that are cellular and over 50% porous in nature, and ISO 1209:2 would be the better alternative, but this standard required samples that were not feasible to manufacture on our FDM printer. To this point, no studies have been found to have followed the appropriate ISO/ASTM standard for scaffold testing; as such, the same original ISO 178 standard used for solid bodies C1 was utilized for the scaffolds S1 and S2.

The plotted results for all three interventions showed primarily the difference in *y*-axis scale (denoting a different range stress on the material), making S1 much less resistant to flexural stimuli; therefore more flexible (Figure 8a–c). This increasingly flexible scaffold allowed testing to the maximum confines of the machine (up to 20 mm of displacement) without fracture of the construct. This result was due to the porous structure of the material and its associated flexibility.^58^ The functionalized S2 behaved in a very similar way, the titanium oxide coating itself having no adverse effect on the flexural properties of this intervention. In both cases, the maximum downward displacement of the Bose machine (20 mm) was insufficient to fracture the scaffold sample due to its inherent flexibility. In contrast, the same displacement enough to fracture the solid sample C1 which exhibited a higher resistance to the applied load. This higher resistance made the solid sample more brittle; it could resist the displacement to a higher degree but once its flexural limit was reached, it fractured. These findings are consistent with previous research^59^ which reported an increase of almost 40% in flexibility when transitioning from a full solid to a 70% porous structure; reinforcing the trend observed in the present study.

Cracks observed in a 3-point bend test can originate from either tension or compression. Compression cracks typically form at the point where the force is applied perpendicularly while tension cracks develop on the opposite, or inferior side of the applied force. This is due to a positive Poisson’s ratio behavior, which results in the material expanding laterally with a perpendicular axial compression.^60^ This behavior was observed in both the solid and scaffold PEEK, but due to the spaces within the porous scaffold, the force was not enough to break the construct as a whole. In fact, because the PEEK scaffold possesses mechanical properties that are not observed in solid PEEK, it can be considered a metamaterial.^61^ Flexurally, both scaffolds with a modulus of 0.21 GPa,(S1) and 0.20 GPa (S2) can ultimately be used to replace trabecular bone which has a modulus of 0.05–0.5 GPa. This ‘trabecular’ infill can then be coated with a perimeter of solid PEEK (C1), which was found to have a flexural modulus of 2.37 GPa to replace cortical bone, which has a flexural modulus of 7–30 GPa.^62^

The fabricated and characterized scaffold shows great promise, mechanically and physically, for use in bone grafting situations. Although PEEK, both filled and unfilled, is already a material used in the armamentarium of osteology, its limited bioactivity has hindered its widespread use especially when it comes to replacing bone. To help amend this, the fabricated scaffold in PEEK needed optimal physical, mechanical and biological properties. In fact, the scaffold produced in this study has increased wettability due to its porous geometry, with pore sizes suitable for infiltration of bone cells. Furthermore, both the unfilled scaffold and the upgraded titanium oxide coated PEEK are not only physically suitable for bone grafting, but also mechanically appropriate, as their flexural properties fall within the range of human bone. Lastly, the titanium oxide coating has been shown in the literature to reduce the formation of fibrous tissue around implants *in vivo*, enhancing the scaffold’s biological suitability. Taken together, these properties make the fabricated scaffold a promising alloplastic alternative for the restoration of critical size bone defects.

## 5 Conclusions

This study demonstrates the successful fabrication of a Titanium-oxide coated, porous PEEK scaffold using optimized FDM techniques, achieving a bone-mimetic architecture with mechanical properties suitable for both trabecular and cortical bone substitution. Flexural testing confirmed a significant increase in flexibility for both the neat scaffold (S1) and titanium-oxide coated PEEK (S2) when compared to solid PEEK (C1). Improved wettability and porosity further support the scaffold’s potential to promote cell adhesion and tissue integration. These findings highlight the scaffold as a promising candidate for bone grafting applications, particularly in load sharing and anatomically complex regions where both mechanical performance and structural adaptability are critical. Future studies will aim to provide comprehensive biological validation, ensuring the construct's safety and efficacy to ensure to ultimately confirm its clinical viability.

## Supplementary Material



## Notes

Atomic force microscopy data obtained from the TiO_2_ coated PEEK; SEM images obtained at three different sites on the TiO_2_ coated PEEK (Figures S2A, S2C, and S2E); EDS data obtained at the same three sites (Figures S2B, S2D, and S2F); EDS tables obtained at the same three sites (Tables S1, S2 and S3) (PDF)
