# Supplementary material for: Fabrication and Characterization of a Porous TiO2‑Modified PEEK Scaffold with Enhanced Flexural Compliance for Bone Tissue Engineering
Source: ACS Biomater Sci Eng. 2025 Sep 23;11(10):5838–49. doi: 10.1021/acsbiomaterials.5c01032 (PMC12522093; doi:10.1021/acsbiomaterials.5c01032)
Supplement: Supplementary file 1 [file ab5c01032_si_001.pdf]

Supporting information for publication:

Fabrication and Characterisation of a Porous TiO2 – modified PEEK Scaffold with Enhanced Flexural Compliance for Bone Tissue Engineering

Martina Galea Mifsud[1], Andrew Sachan[2], Roger J. Narayan [2], Lucy Di-Silvio[1]‡, Trevor Coward[1]‡*

*Corresponding author – Can be contacted on k20121335@kcl.ac.uk

‡ These authors contributed equally.

[1]Faculty of Dentistry, Oral & Craniofacial Sciences, King's College London, London SE1 9RT, UK

[2]Joint Department of Biomedical Engineering, University of North Carolina and Carolina State University, Raleigh, North Carolina 27695-7115, United States

---

Number of pages: 9

Number of figures: 10

Number of tables: 3

The atomic force microscopy data were obtained from the titanium coated polyetheretherketone (PEEK) scaffold using a nanoIR3 instrument (Bruker, Billerica, MA, USA) that was operated in tapping mode with a 300 kHz tip. To acquire the atomic force microscopy data, the image resolution was 256 x 256, and the scan rate was 0.5 Hz for a 10 µm x 10 µm image. The images were flattened via a first-order flatten using the Analysis Studio software (Bruker, Billerica, MA, USA). The images and corresponding root mean square (RMS) roughness values obtained from the sample are provided below. Data was acquired from one location on the sample. A 5 µm x 5 µm image and a 10 µm x 10 µm image were obtained as follows:

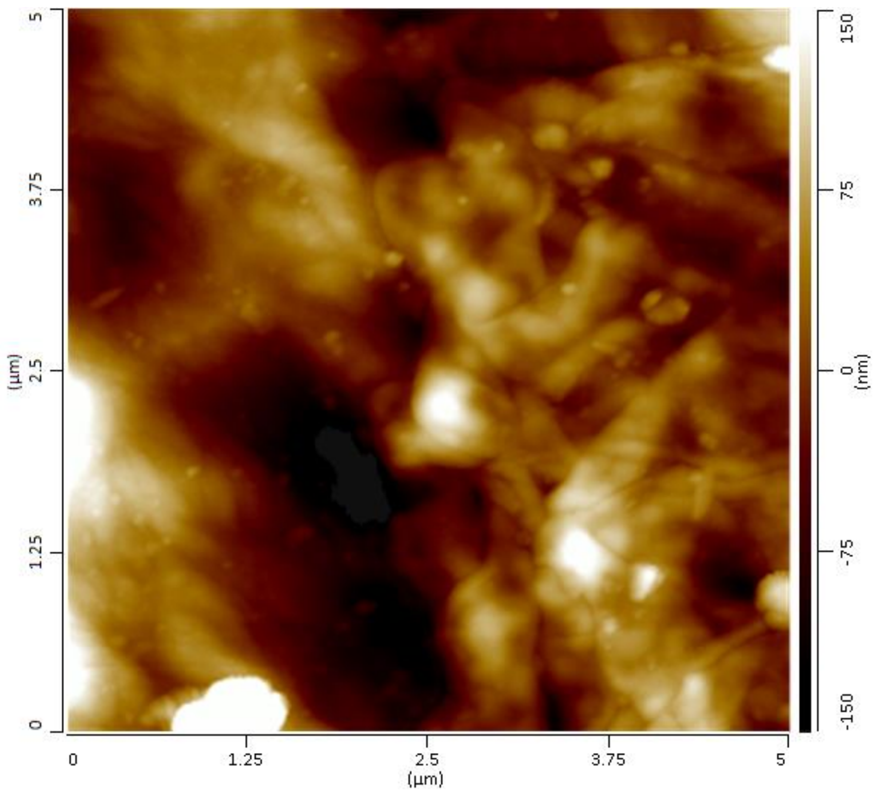

Figure S1A. 5 µm x 5 µm AFM image. The RMS roughness corresponding to the image is 62.67 nm.

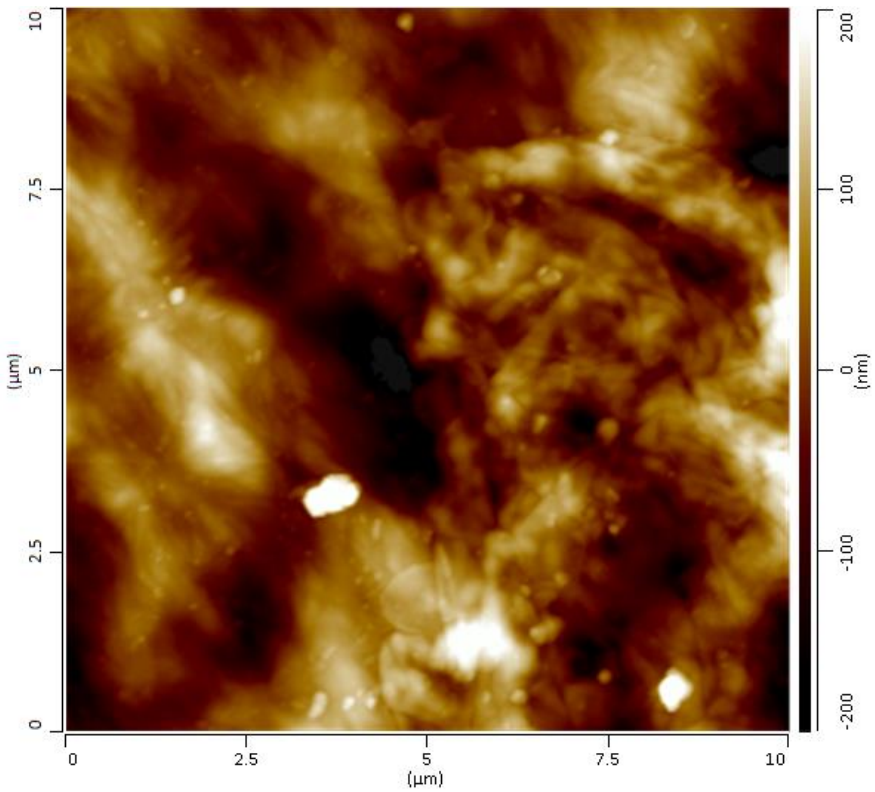

Figure S1B. 10 µm x 10 µm AFM image. The RMS roughness corresponding to the image is 73.31 nm.

Data were subsequently acquired from another location on the sample. A 5 µm x 5 µm image and a 10 µm x 10 µm image were obtained as follows:

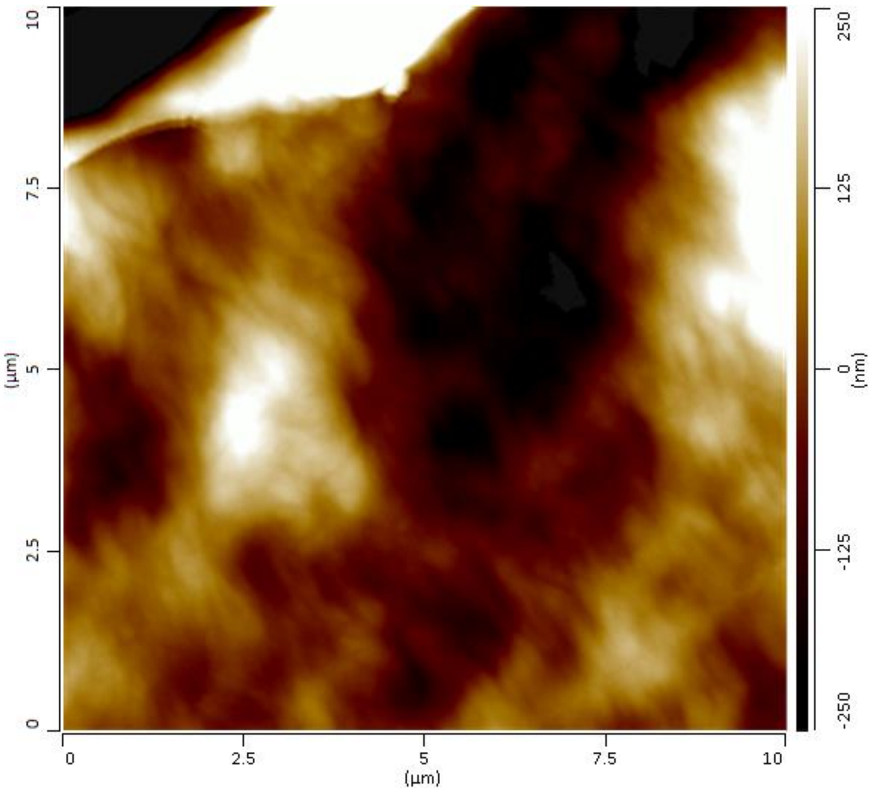

Figure S1C. 5 µm x 5 µm AFM image. The RMS roughness corresponding to the image is 165.25 nm.

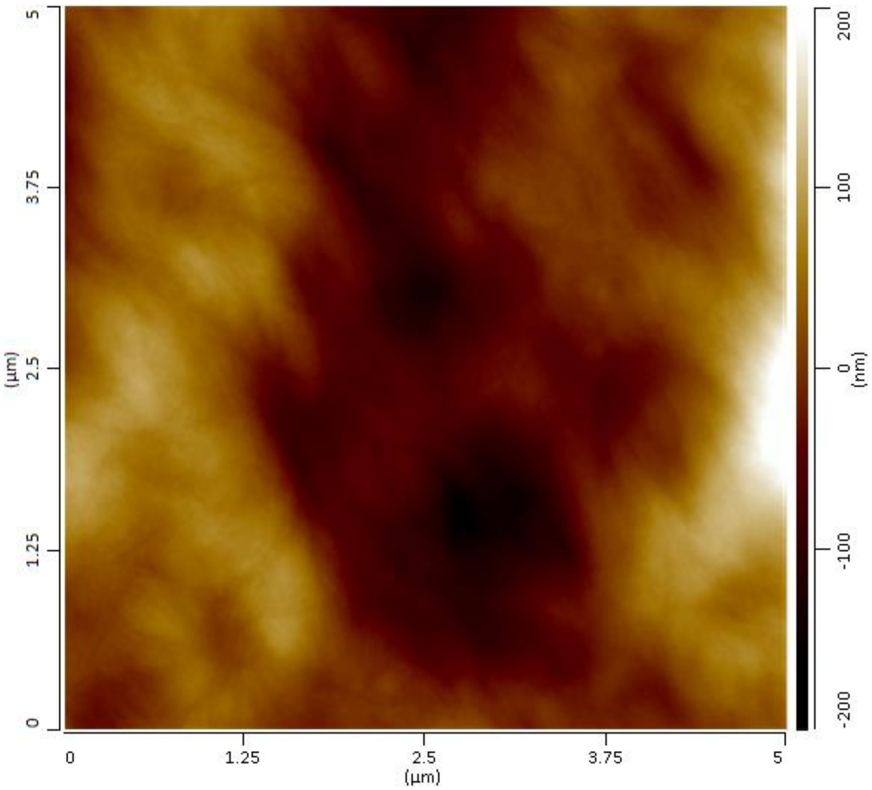

Figure S1D. 10 µm x 10 µm AFM image. The RMS roughness corresponding to the image is 61.70 nm.

Scanning electron microscopy (SEM) and energy dispersive spectroscopy (EDS) data were acquired from the titanium coated PEEK scaffold using a S-4700 field emission microscope (Hitachi, Tokyo, Japan). The scaffold was attached using a double-stick carbon tape to an aluminium sample holder. The coated scaffold was not coated with AuPd alloy prior to analysis. The operating conditions included an accelerating voltage of 2 kV, a beam current of 10 μA, and a working distance of close to 12 mm; these conditions were maintained throughout the analysis activities. The EDS data were obtained using an INCA PentaFet-X3 instrument (Oxford Instruments, Abingdon, Oxfordshire, England) microscope that was operated at an acceleration voltage of 20 kV; the EDS instrument was attached to the microscope.

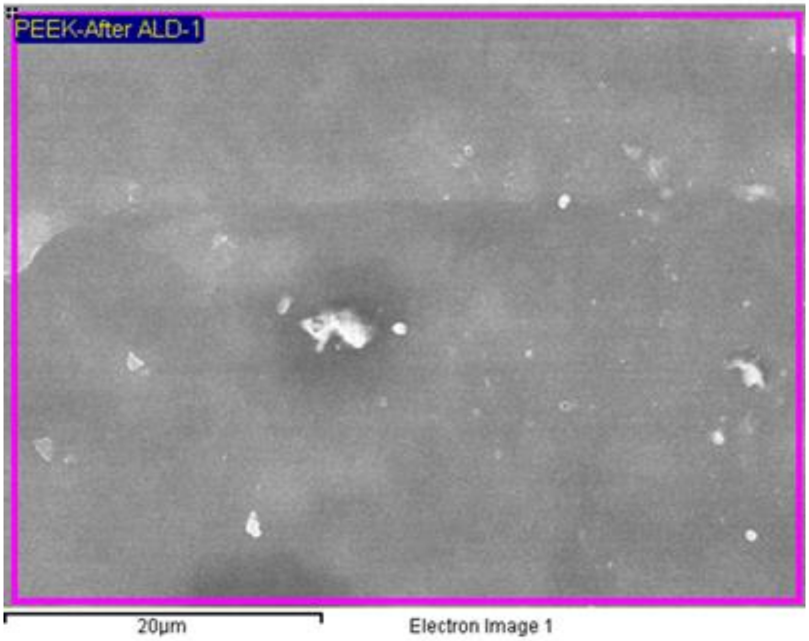

Figure S2A. SEM image associated with Site #1 on the titanium coated PEEK scaffold.

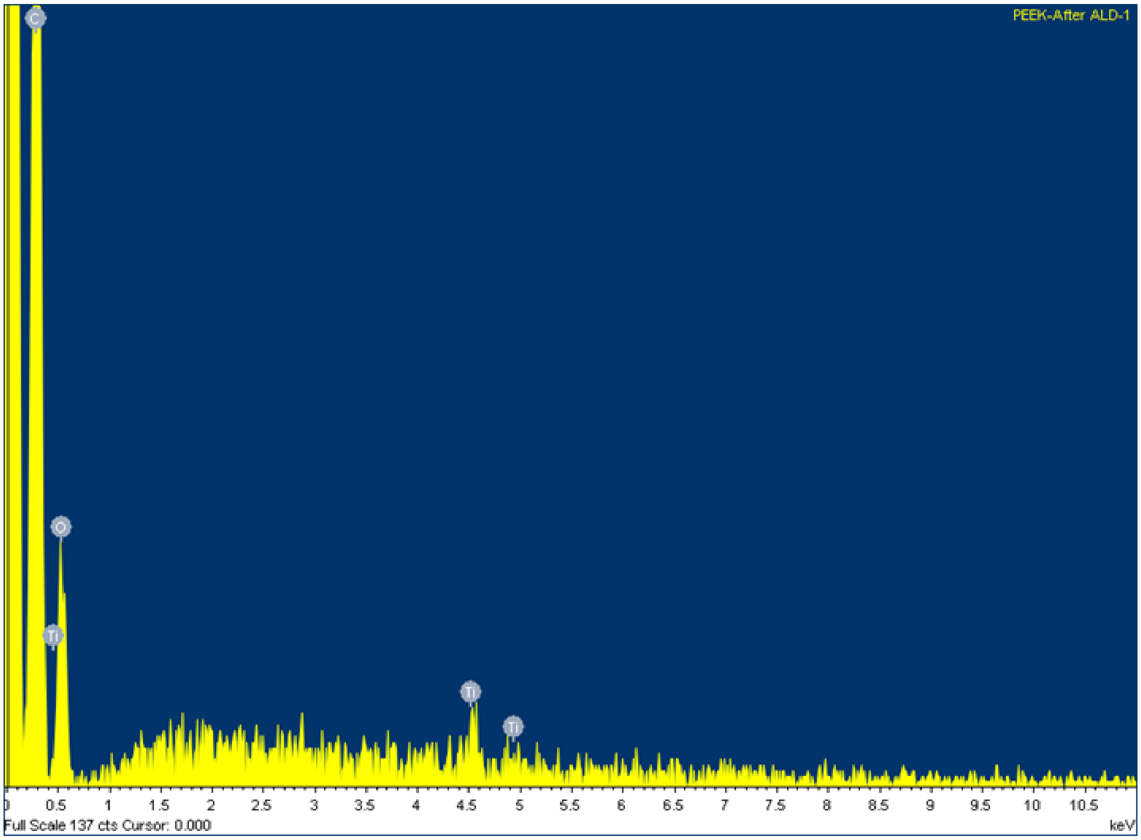

Figure S2B. EDS data associated with Site #1 on the titanium coated PEEK scaffold.

Table S1. EDS data showing the presence of various elements associated with Site #1 on the titanium coated PEEK scaffold.

Element	App Conc.	Intensity Corrn.	Weight%	Weight% Sigma	Atomic%
C K	13.10	1.4691	71.57	3.74	77.73
O K	1.15	0.3452	26.76	3.80	21.82
Ti K	0.17	0.8010	1.67	0.43	0.46
Totals			100.00		

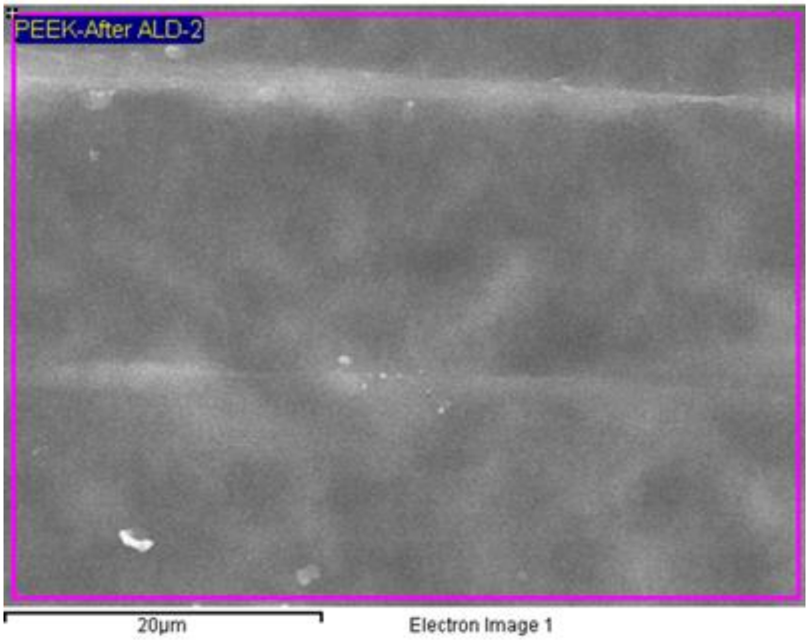

Figure S2C. SEM image associated with Site #2 on the titanium coated PEEK scaffold.

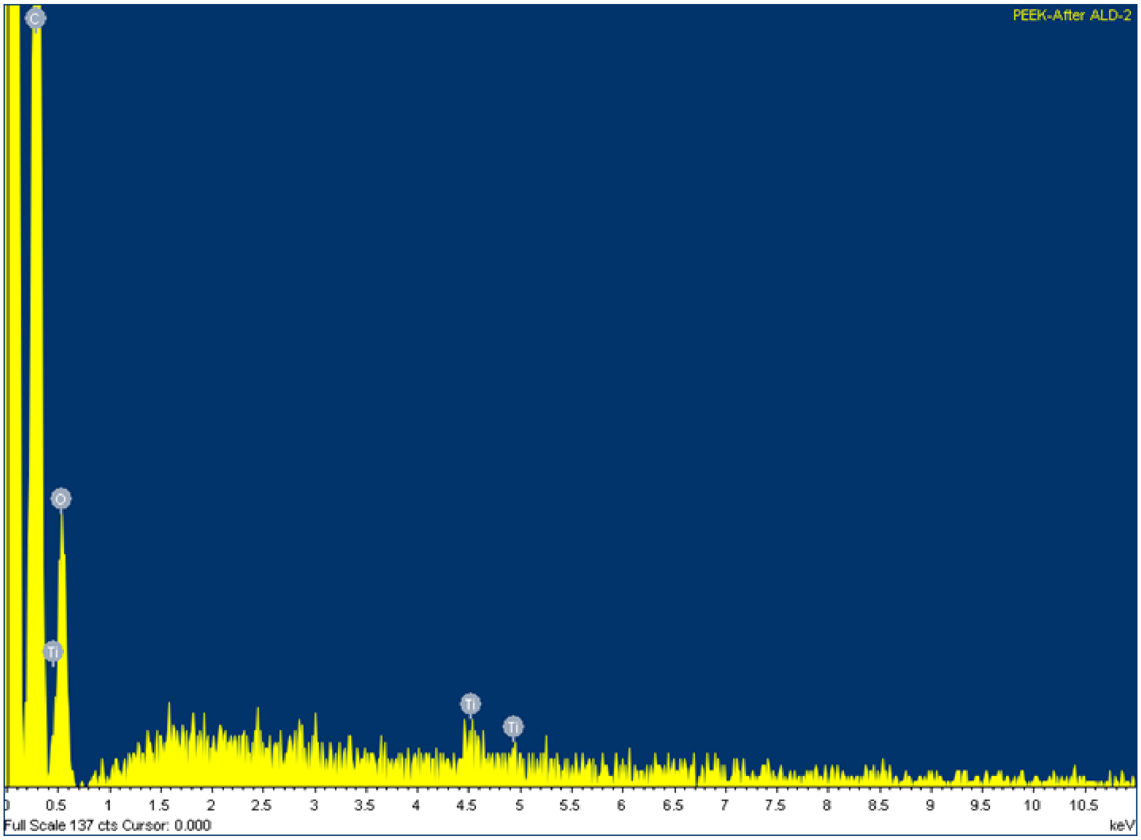

Figure S2D. EDS data associated with Site #2 on the titanium coated PEEK scaffold.

Table S2. EDS data showing the presence of various elements associated with Site #2 on the titanium coated PEEK scaffold.

Element	App Conc.	Intensity Corrn.	Weight%	Weight% Sigma	Atomic%
C K	13.22	1.4766	71.79	3.96	77.63
O K	1.19	0.3501	27.24	3.99	22.11
Ti K	0.10	0.7999	0.97	0.43	0.26
Totals			100.00		

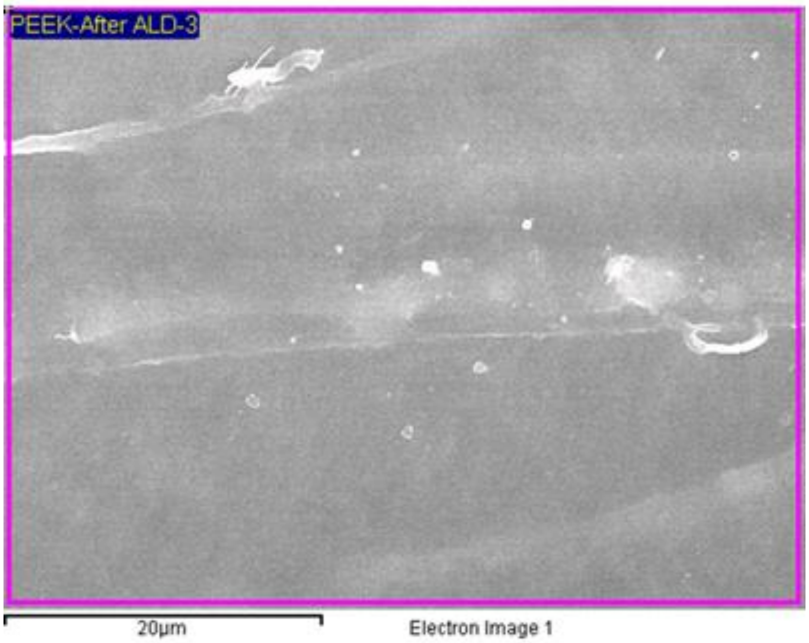

Figure S2E. SEM image associated with Site #3 on the titanium coated PEEK scaffold.

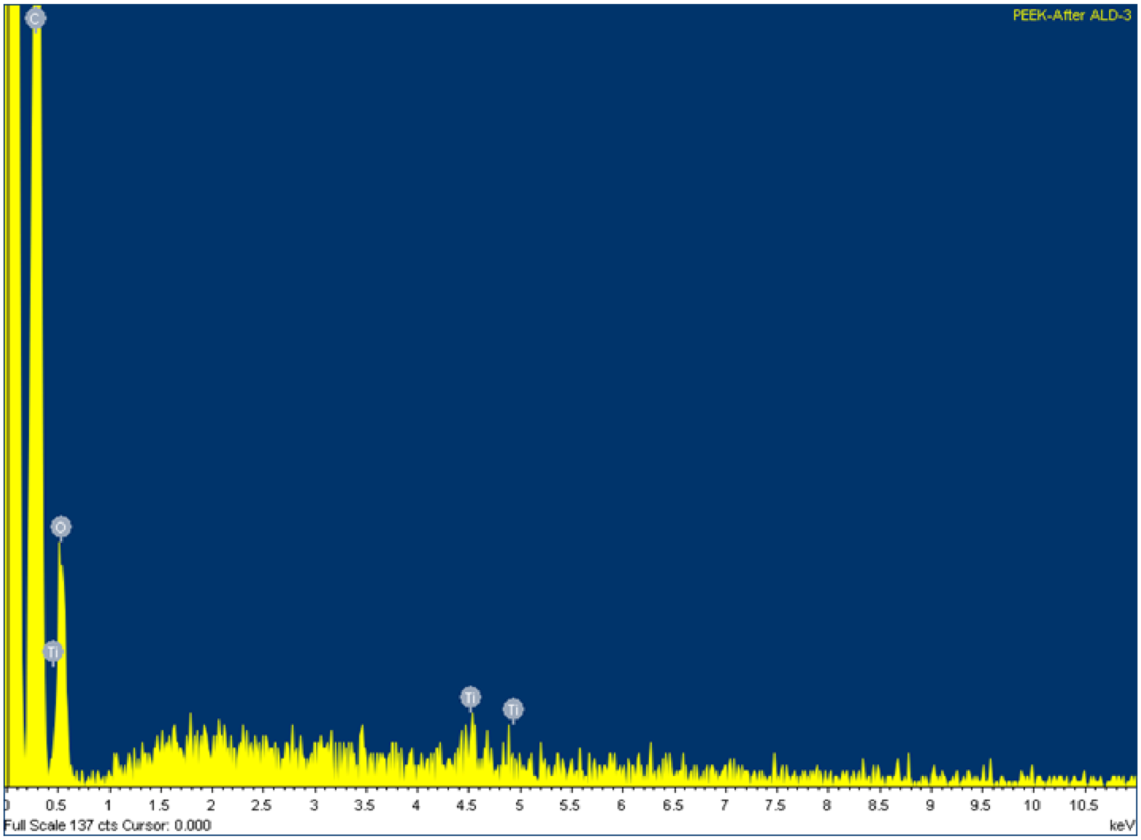

Figure S2F. EDS data associated with Site #3 on the titanium coated PEEK scaffold.

Table S3. EDS data showing the presence of various elements associated with Site #3 on the titanium coated PEEK scaffold.

Element	App Conc.	Intensity Corrn.	Weight%	Weight% Sigma	Atomic%
C K	13.02	1.4630	70.98	3.55	76.87
O K	1.26	0.3556	28.17	3.57	22.90
Ti K	0.09	0.7999	0.85	0.42	0.23
Totals			100.00		
